# Increasing bladder capacity by foot stimulation in rats with spinal cord injuries

**DOI:** 10.1186/s12894-017-0277-4

**Published:** 2017-09-15

**Authors:** Guoqing Chen, Limin Liao, Zhaoxia Wang, Xing Li, Wenjuan Du

**Affiliations:** 1 Department of Urology, China Rehabilitation Research Center, Beijing, 100068 China; 2 Department of Urology, Capital Medical University, Beijing, China

**Keywords:** Foot stimulation, Spinal cord injury, Rat, Cystometrogram, Bladder capacity

## Abstract

**Background:**

This study was to explore the possibility that foot stimulation increased bladder capacity(BC) in rats with neurogenic bladder secondary to T10 spinal cord injuries.

**Methods:**

In 20 awake rats (stimulation group) with T10 spinal cord injuries, 5 repeat cystometrograms (CMGs) were recorded. The 1st and 2nd CMGs were performed without stimulation. The 3rd, 4th, and 5th CMGs were done separately with 1 T, 2 T, and 4 T stimulation, respectively, through a pair of pad electrodes on the skin of the hind foot. In the control group of 20 rats, 5 repeat CMGs were recorded without foot stimulation. The threshold (T) was the minimal stimulation intensity to induce an observable toe twitch.

**Results:**

In the stimulation group, foot stimulation with 2 T significantly increased the BC an additional 68.9% ± 20.82% (*p* < 0.05). Foot stimulation with 4 T increased the BC an additional 120.9% ± 24.82% (*p* < 0.05). Compared with the control group, BC in the 1st, 2nd, and 3rd (1 T) CMG had no significant difference in the stimulation group, but the 4th (2 T) and 5th (4 T) CMGs were significantly increased (p < 0.05).

**Conclusions:**

Electrical stimulation of the foot was effective in inhibiting reflex bladder activity and increasing bladder capacity in spinal cord injury rats.

## Background

In the supra-sacral spinal cord injury (SCI) patients, the loss of supraspinal control leads to neurogenic detrusor overactivity (NDO) and detrusor sphincter dyssynergia (DSD), which can result in small bladder capacity、high bladder pressure during micturition, vesicoureteral reflux and renal impairment. The target of treatment must meet the following three points: urine storage with low pressure, voiding with low pressure and adequate bladder emptying [1]. Clean intermittent catheterization (CIC) with muscarinic receptor antagonists is the gold standard treatment for neurogenic lower urinary tract dysfunction (NLUTD), but the drug has a few side effects which prompt urologists to select new therapeutic method [2].

Recently, sacral neuromodulation (SNM) [3], pudendal nerve stimulation (PNS) [4]and tibial nerve stimulation (TNS) [5] are considered very valuable for the treatment of NDO secondary to SCI; but, SNM and PNS need to implant an electrode and an stimulator, and TNS need to insert a needle electrode near to the nerve.

Stimulation of the foot using skin surface electrodes is a new, non-invasive, easily accessible, convenient way to inhibit bladder activity and increase bladder capacity, which has been verified in cats with overactive bladders [6] and healthy humans [7]. In our previous study, we reported that electrical stimulation of somatic afferent nerves in the feet can increase bladder capacity in patients with neurogenic bladders after sigmoid cystoplasty [8]. Although a positive effect was shown in those patients, we need to do further research in patients with other types of neurogenic bladder.

In this study we explored the possibility that foot stimulation with surface electrodes increased bladder capacity in rats with neurogenic bladder secondary to T10 SCI.

## Methods

### Ethics statement

All protocols involving the use of animals in this study were approved by the Ethics Committee of Capital Medical University, China.

### Animal model

A total of 40 adult female Sprague-Dawley rats weighing 236–270 g were used. To produce a SCI, the rats were anesthetized with pentobarbital (30 mg/kg i.p.). We performed T10 laminectomy and open the dura of the rats, transected the T10 spinal cord with scissors and placed an absorbable gelatin sponge between the cut ends, then sutured the muscle and skin. Rats were then put on an electric warmer to maintain body temperature until recovering from anesthesia. SCI animals were treated post-operatively with ampicillin (100 mg/kg i.m.) for 5 days. As we known, detrusor areflexia persist during spinal shock period after suprasacral spinal cord injury, so the bladder need to be emptied by abdominal compression twice a day until reflex detrusor contraction recovered, often 10–14 days after surgery. Then SCI rats were divided into two groups: 1) control group, SCI rats without foot stimulation (*n* = 20); and 2) stimulation group, SCI rats with foot stimulation (n = 20).

### Surgical procedure for experiments

Three weeks after SCI when reflex detrusor contraction recovered, the animals in both groups were anesthetized with 2% isoflurane, and the abdomen was opened via a midline laparotomy. The ureters were cut and drained externally and the urethra was secured by a ligature. A PE-90 catheter was inserted into the bladder for recording the intravesical pressure through the bladder dome. The catheter was connected to a pressure transducer (MP150; Biopac, Goleta, CA, USA) and micro-infusion pump (Stoelting, Wood Dale, IL, USA) through a three-way stopcock. After the abdomen was closed, the rats were placed in a restrainer and were allowed to recover from anesthesia for 1–2 h. After removing the fur, a pair of surface self-adhesive pad electrodes (Grass F- E10ND, diameter = 1 cm; Astro-Medical Inc., Mentor, OH, USA) were attached to the skin area on both hind feet. A Grass S88 stimulator (Grass Medical Instruments, West Warwick, RI, USA) with stimulus isolator (SIU5; Grass Medical Instruments) was used to generate stimulus pulses.

**Experiment**: Cystometry in an awake condition (*n* = 20 each of two groups).

After the rats recovered from anesthesia, the bladder was infused with physiologic saline at a rate of 0.08 ml/min until repetitive detrusor contractions were elicited. After at least two stable micturition cycles were observed, cystometric parameters can be recorded.

In the control group, five cystometrograms (CMGs) were performed without stimulation. In the stimulation group, five CMGs were performed as follows: 1 and 2, control CMG without stimulation; 3, CMG during 1 T stimulation; 4, CMG during 2 T stimulation; and 5, CMG during 4 T stimulation. A 3–5 min rest period was performed after each CMG to allow the detrusor to recover from dilatation. Foot stimulation was done with uniphasic rectangular pulses (5 Hz frequency, 0.2 ms pulse width) according the previous research [6]. The threshold (T) stimulation intensity (3–16 V), which was defined as the minimal intensity to induce an observable toe twitch, was determined by slowly increasing the stimulation intensity at the beginning of the experiment.

The bladder capacity (BC) was defined as the bladder volume threshold to induce a bladder reflex contraction of large amplitude (>30 cmH_2_O) and long duration (>20 s) [9, 10].

### Statistical analysis

Statistical analysis was performed using graph pad prism 6.0. For the repeated CMG recordings, BC was normalized to the initial saline control capacity in the same animal to allow comparisons between animals. Capacity measurements under the same conditions were averaged and reported as the mean ± standard error of the mean. Statistical significance (*P* < 0.05) was detected by ANOVA, followed by Dunnett or Bonferroni post-tests.

## Results

**Fig. 1:**
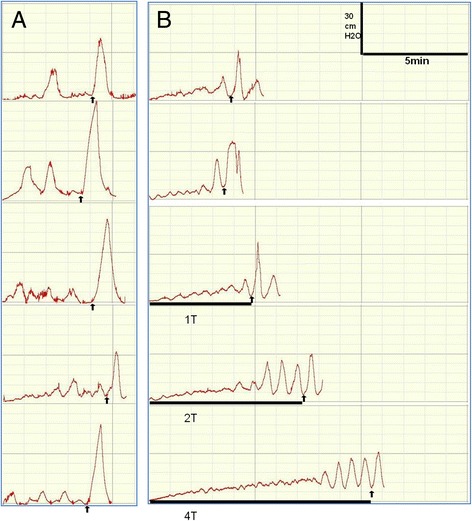
**a**: In control group, bladder capacity was not significantly changed during repeated CMGs in the absence of foot stimulation. **b**: In treatment group, the 1st and 2nd was the BC without stimulation. The 3rd, 4th and 5th were the BC with 1 T, 2 T and 4 T stimulation. The arrows indicatethe stop of bladderinfusion. The black bars under the pressure traces indicate the stimulation duration

**Fig. 2:**
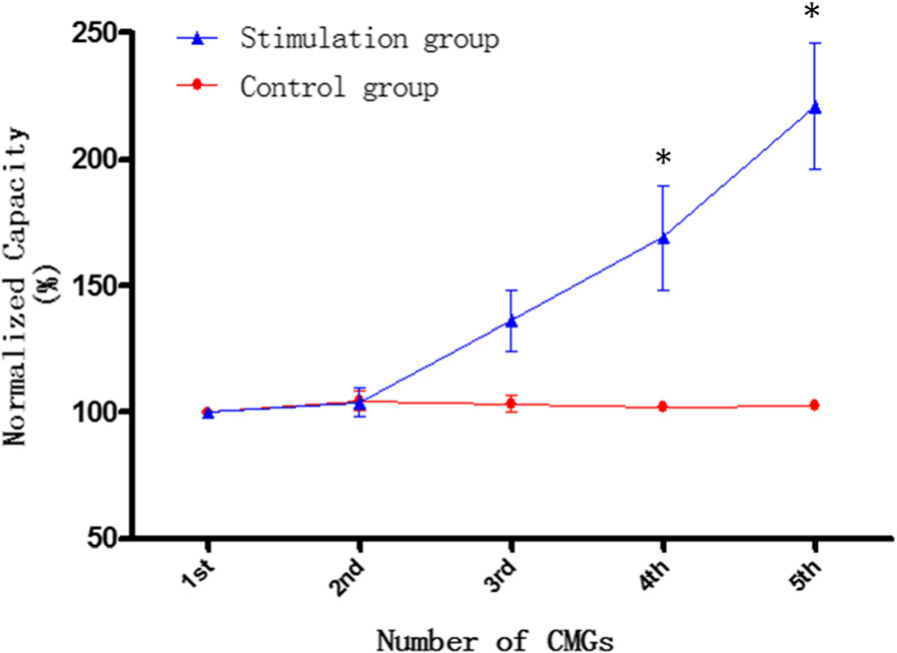
BC in control and stimulation group. Asterisk indicates control vs treatment statistically significantly different (*p* < 0.05). In treatment group, the 1st and 2nd was the BC without stimulation. The 3rd、4th and 5th were the BC with 1 T、2 T and 4 T stimulation. In control group, all the five BC were not with stimulation. T was stimulation intensity (3–16 V)

In the control group, BC did not differ significantly in five repeat continuous CMGs (*p* > 0.05) (Fig. 1a). In the stimulation group (Fig. 1b), the 1st and 2nd CMGs without stimulation were not significantly different with respect to BC (*p* > 0.05). Although foot stimulation with 1 T increased BC an additional 36.2% ± 11.75%, this difference was not significantly different compared with the 1st and 2nd CMG (p > 0.05). Foot stimulation with 2 T significantly increased BC an additional 68.9% ± 20.82% (*p* < 0.05). Foot stimulation with 4 T significantly increased BC an additional 120.9% ± 24.82% (*p* < 0.05). Compared with the control group (Fig. 2), BC in the 1st, 2nd, and 3rd (1 T) CMG did not differ significantly in the stimulation group, but the 4th (2 T) and 5th (4 T) CMG increased significantly (*p* < 0.05).

## Discussion

Neurogenic bladder (NB) dysfunction after SCI is a major medical and social problem. The ultimate goal of any urologic treatment is to prevent upper urinary tract damage and renal failure [1]. Currently, the gold standard treatment for NDO is clean intermittent catheterization (CIC) combined with muscarinic receptor antagonists; however, many patients are often refractory to the medication because of side effects [2]. So somatic nerve stimulation was proposed to treat bladder dysfunction for complement medication in recently [11].

Previous studies have shown that foot stimulation with surface electrode can inhibit reflex micturition, significantly increase BC, and induce post-stimulation inhibition of reflex bladder activity that persists for 1–2 h in cats [6]. Actually, the same effect can be found in healthy volunteers [7]. It showed that foot stimulation with surface electrode can delay bladder filling sensation and significantly increase BC > 50% in healthy humans.

In our previous study [8], we found that foot stimulation delayed the bladder filling sensation and significantly increased BC in the patients after bladder augmentation. Comparing with baseline, the volume per CIC was significantly increased, and this long-lasting effect persisted nearly 1 day. This is the first clinical trial in which electrical stimulation of the foot was used to treat patients. Although a positive effect was shown in the current study, the patients did not represent all types of lower urinary tract disorders.

Thus, in this study we used BC to evaluate the effect of foot stimulation on bladder function by CMG in rats secondary to T10 spinal cord injury. In the control group, BC did not differ significantly in the five continuous CMGs which means repeated CMG did not increase BC. In the stimulation group, the 1st and 2nd CMGs without stimulation did not differ significantly. Although foot stimulation with 1 T increased the BC an additional 36.2% ± 11.75%, this difference was not significant (*p* > 0.05). Foot stimulation with 2 T significantly increased the BC an additional 68.9% ± 20.82% (*p* < 0.05). Foot stimulation with 4 T significantly increased the BC an additional 120.9% ± 24.82% (*p* < 0.05). Compared with the control group, this enlargement was the effect of foot stimulation, but not repeated bladder infusion.

The mechanism underlying foot stimulation is unknown, but may be mediated by the nerves in the foot [6–8]. The stimulation electrodes were placed on the skin surface rather than directly on the nerves. Which nerves were activated? The tibial nerve courses from the inner ankle inferiorly to the plantar surface of the foot and branches into the lateral and medial plantar nerves at the location of the electrodes. These nerves further branch into multiple small nerves that course toward the toes. Thus, it is highly likely that foot stimulation activates afferent branches of the tibial nerve in the lateral and medial plantar aspects of the foot.

A previous study in cats showed that the inhibitory effect on BC elicited by electrical stimulation of the nerves from the hind limb muscles was lost after chronic spinal cord transection at the thoracic level, indicating a possible role of the supra-spinal mechanisms in somatovesical inhibition [12]. In the current study, the complete T10 SCI model was used. The previous research showed sacral neuromodulation (SNM) [3], pudendal nerve stimulation (PNS) [4], and tibial nerve stimulation [5] all inhibit bladder overactivity in complete SCI patients. Thus, we suggest that foot stimulation should inhibit detrusor overactivity and increase BC in complete SCI patients as in animal models. It is highly likely that foot stimulation activates afferent branches of the tibial nerve in the lateral and medial plantar aspects of the foot.

In this study, 4 T stimulation achieved a good result in BC, but the foot twitched strongly under this intensity. If we use this intensity in patients in the future, it may induce discomfort in patients. Thus, we will investigate whether or not low-intensity stimulation combined with low-dose drug can increase BC as high-intensity stimulation, but no adverse event occurred which induced by high-intensity stimulation and high-dose drug.

## Conclusions

Foot stimulation with surface electrode was effective to inhibit reflex bladder activity and increase BC in SCI rats. These results showed that electrical stimulation of the foot might be an effective treatment for neurogenic bladder secondary to SCI.

## Abbreviations

BC: Bladder capacity
CIC: Clean intermittent catheterization
CMGs: Cystometrograms
DSD: Detrusor sphincter dyssynergia
NDO: Neurogenic detrusor overactivity
NLUTD: Neurogenic lower urinary tract dysfunction
PNS: Pudendal nerve stimulation
SCI: Spinal cord injury
SNM: Sacral neuromodulation
T: Threshold
TNS: Tibial nerve stimulation
